# Depression and Anxiety Among Young Gender- and Sexuality-Diverse Adolescents

**DOI:** 10.1001/jamanetworkopen.2025.51570

**Published:** 2025-12-29

**Authors:** Sarita Bista, Aliza Werner-Seidler, Kate Maston, Ashleigh Lin, Yael Perry, Cristyn Davies, Petra L. Graham, Helen Christensen, Jennifer L. Marino, S. Rachel Skinner

**Affiliations:** 1 Specialty of Child and Adolescent Health, Children’s Hospital Westmead Clinical School, Faculty of Medicine and Health, The University of Sydney, Westmead, New South Wales, Australia; 2 Black Dog Institute, University of New South Wales, Sydney, New South Wales, Australia; 3 School of Population and Global Health, University of Western Australia, Perth, Western Australia, Australia; 4 Australian Research Centre in Sex, Health and Society, La Trobe University, Bundoora, Victoria, Australia; 5 The Kids Research Institute Australia, University of Western Australia, Perth, Western Australia, Australia; 6 School of Social Sciences, Western Sydney University, New South Wales, Australia; 7 School of Mathematical and Physical Sciences, Macquarie University, Macquarie Park, New South Wales, Australia; 8 The Children’s Hospital at Westmead, Westmead, New South Wales, Australia

## Abstract

**Importance:**

Gender- and sexuality-diverse (GSD) youths are at increased risk of depression and anxiety compared with their cisgender and heterosexual counterparts. Little is known about the risks for younger GSD adolescents (<15 years).

**Objective:**

To estimate the prevalence and odds of depression and anxiety for younger GSD adolescents.

**Design, Setting, and Participants:**

This cross-sectional study collected baseline data across 3 separate cohorts of year (grade) 8 students (August through September 2019; August through November 2020; and April 2021 through March 2022) in the Future Proofing Study, an Australian prospective study. All year 8 students from 134 secondary participating schools across Australia were invited to participate in the survey. Data were analyzed in April 2025.

**Exposures:**

Gender diversity and sexuality diversity status.

**Main Outcomes and Measures:**

Self-reported depression symptoms were assessed using the Patient Health Questionnaire for Adolescents, and anxiety symptoms were assessed using the Spence Children’s Anxiety Scale.

**Results:**

The sample comprised 6388 adolescents (mean [SD] age, 13.9 [0.5] years); 5842 (91.5%) were born in Australia, 5982 (93.7%) spoke English at home, and 5009 (78.4%) lived in a 2-parent home. Sex at birth included 3329 (52.1%) female, 2968 (46.5%) male, 33 (0.5%) unsure, 51 (0.8%) prefer not to report, and 6 (0.1%) another. There were 6019 cisgender respondents (95.0%), 209 gender-diverse respondents (3.3%), and 109 prefer not to report about gender (1.7%); sexuality identity included 4472 heterosexual respondents (70.0%), 767 sexuality-diverse respondents (12.0%), 559 respondents (8.8%) unsure, 296 prefer not to report (4.6%), and 294 respondents (4.6%) missing these data. Of the 6388 adolescents in the sample, 3745 (58.6%) reported depression (2780 [43.5%] mild to moderate, 965 [15.1%] clinical levels), and 1888 (29.6%) reported anxiety (958 [15.0%] mild to moderate, 930 [14.6%] clinical levels). Larger proportions of GSD adolescents reported clinical levels of depression (gender diverse: 59.3% vs 13.3%, *P* < .001; sexuality diverse: 43.4% vs 10.1%, *P* < .001) and anxiety (gender diverse: 48.8% vs 13.1%, *P* < .001; sexuality diverse: 35.3% vs 10.9%, *P* < .001) than cisgender and heterosexual counterparts, respectively. Larger proportions of GSD adolescents faced greater demographic individual, psychosocial, and contextual adversities. In multivariable multinomial logistic models, GSD status was associated with higher odds of clinical-level depression (gender-diverse odds ratio [OR], 5.68 [95% CI, 3.46-9.33]; sexuality-diverse OR, 6.49 [95% CI, 4.66-9.03]) and anxiety (gender-diverse OR, 3.49 [95% CI, 2.46-4.95]; sexuality-diverse OR, 3.07 [95% CI, 2.40-3.93]) compared with cisgender and heterosexual peers.

**Conclusions and Relevance:**

In this cross-sectional study, higher prevalence and risk of greater depression and anxiety symptoms were evident among younger GSD adolescents. To improve their mental health, prevention and interventions addressing identity-specific stressors and broader contextual risk and protective factors should be initiated by early adolescence.

## Key Points

**Question:**

What are the prevalence rates and odds of depression and anxiety for younger gender- and sexuality-diverse adolescents (<15 years of age)?

**Findings:**

In this cross-sectional study of 6388 younger adolescents, gender and sexuality diversity status was associated with depression and anxiety, with 5 and 6 times higher odds of clinical levels of depression for gender-diverse and sexuality-diverse adolescents, respectively, and 3 times higher odds of clinical-level anxiety for both groups, compared with their cisgender and heterosexual counterparts.

**Meaning:**

Mental health interventions and prevention strategies targeting identity-specific stressors and broader contextual risks in early adolescence are urgently needed.

## Teaser

This cross-sectional study estimates the prevalence rates and odds of depression and anxiety among gender- and sexuality-diverse adolescents younger than 15 years of age in Australia.

## Introduction

While mental health challenges are common in adolescence, gender- and sexuality-diverse (GSD) adolescents face disproportionately higher rates of depression and anxiety than their cisgender and heterosexual peers.^1,2,3,4,5,6,7,8^ These disparities are explained by minority stress theory, suggesting stigma, discrimination, and victimization increase risks for psychopathology,^9^ and by shared general psychological processes (eg, coping, emotional regulation, social/interpersonal, and cognitive processes) across groups, which shape developmental trajectories of psychopathology, with stressors faced by GSD adolescents associated with differences from cisgender heterosexual peers.^10^ An integrated framework^11^ suggests that stigma-related stress elevates psychological processes that mediate the association between stigma-related stress and psychopathological outcomes.^11^ Evidence shows that GSD adolescents are particularly vulnerable to experiencing discrimination, bullying, social exclusion, family rejection,^3,12,13,14,15,16,17,18,19,20,21^ emotional dysregulation,^22,23,24^ and negative school climate and connectedness,^25^ heightening depression and anxiety risks. Transgender (trans) and gender-diverse adolescents face added burdens from navigating identity and societal expectations, scrutiny, discrimination, and limited access to affirming or inclusive health care.^4,26,27^ Conversely, family and peer support and positive school climate and connection act as protective factors, lowering mental health risks for GSD adolescents.^18,19,21,28,29,30^ These findings indicate that mental health disparities arise from intersecting minority stress, psychosocial processes, and environmental factors. However, there is limited evidence about the mental health outcomes of the GSD population accounting for these intersecting factors concurrently. Furthermore, robust research remains limited by smaller samples of GSD adolescents, focus on a single subgroup, or diverse identities, such as lesbian, gay, bisexual, transgender, queer, questioning, and other, collapsed into LGBTQ+ and often excluding persons unsure or declining to report their gender and sexuality identities, despite heightened mental health risks in these groups.^5,31^ Additionally, studies of younger GSD adolescents (<15 years) in representative samples are scarce.

This study investigated the prevalence and risks of depression and anxiety symptoms in younger GSD adolescents, including those who prefer not to report or are unsure of their sexuality or gender, with cisgender and heterosexual as comparison groups. This study considers several demographic, psychosocial, family, and school factors that may confound, or additively or interactively influence, the association between GSD and depression and anxiety.

## Methods

### Study Design, Setting, and Participants

This cross-sectional study was approved by the University of New South Wales (NSW) Human Research Ethics Committee, the State Education Research Applications Process for the NSW Department of Education, and relevant Catholic school dioceses. Written informed consent was obtained from a parent or guardian and the student participant. This report follows the Strengthening the Reporting of Observational Studies in Epidemiology (STROBE) reporting guideline.

This study used cross-sectional baseline data of year (grade) 8 students from the Future Proofing Study (FPS), a prospective cohort study on adolescent mental health in Australian secondary schools. Among all government and nongovernment secondary schools invited across NSW and independent schools in capital cities across Australia, 134 schools participated. All year (grade) 8 students (typically aged 13-14 years) from participating schools were eligible if they owned a smartphone (iOS or Android) with an active number and provided written informed consent from a parent or guardian and the student. Participating schools were required to have an onsite counselor, psychologist, or well-being staff present during data collection. Data were collected across 3 separate school year 8 cohorts: August through September 2019; August through November 2020; and April 2021 through March 2022. Data were analyzed in April 2025. A detailed study design and protocol have been published elsewhere.^32,33^

### Measures

#### Outcomes

##### Depression Severity

Depression severity was measured using the 9-item Patient Health Questionnaire for Adolescents (PHQ-9), with 4-point scale responses: 0 (not at all) to 3 (nearly every day during last 2 weeks) and total scores ranging from 0 to 27. Standard cutoff scores for depression symptoms are 0 to 4 for none or minimal, 5 to 9 for mild, 10 to 14 for moderate, 15 to 19 for moderately severe, and 20 to 27 for severe.^34^ Scores of 15 or higher (moderately severe or severe) indicate clinical-level symptoms.^24,34,35,36^ For analysis, scores were collapsed into categories of 0 for no symptoms (0-4), 1 for mild to moderate symptoms (5-14), and 2 for moderately severe to severe (>15, ie, clinical level symptoms). The PHQ-9 has demonstrated good psychometric properties,^35,37,38^ with high internal consistency (Cronbach α = 0.88) in the FPS data.^33^

##### Anxiety Severity

Anxiety was assessed using the 8-item Spence Children’s Anxiety Scale-Short Form (SCAS-8),^39^ with 4-point scale responses: 0 (never) to 3 (always) and total scores ranging from 0 to 24. Since universal cutoff scores for the SACS-8 are unavailable, we used the cutoff scores determined by large Australian studies of children and adolescents:^39,40^ normal (≤9 for males and ≤11 for females), elevated (≥10 for males and ≥12 for females), and clinical levels of anxiety (≥13 for males and ≥16 for females). For other gender (gender diverse), ≤10, ≥11, and ≥15 are indicative of normal, elevated, and clinical levels of anxiety, respectively.^40^ We applied the male cutoff scores to all male participants (cisgender and gender diverse), the female cutoff scores to all female participants (cisgender and gender diverse), and the other cutoff scores to nonbinary participants, those preferring not to say, and those who were missing gender data. For analysis, scores were categorized as 0 (normal or no symptoms), 1 (elevated or mild to moderate), and 2 (clinical-level symptoms). The SCAS-8 has demonstrated good reliability,^39^ and high internal consistency (Cronbach α = 0.88) in the FPS data.^33^

#### Independent Variables and Covariates

The primary independent variables were self-reported gender diversity and sexuality diversity. Following Australian Bureau of Statistics (ABS) guidelines,^41^ gender diversity was derived using a 2-step method—cross-classifying sex at birth and current gender identity questions—incorporating responses from nonbinary, another term (specified), and other (specified). For analysis, gender diversity was categorized as cisgender, gender diverse, prefer not to report, and missing. The term *unsure* for current gender was unavailable. Sexuality diversity was based on the sexual orientation question (response options: heterosexual or straight, gay or lesbian, bisexual, pansexual, asexual, other (specify), not sure, and prefer not to say), and categorized as: heterosexual, sexuality diverse, prefer not to report, unsure, and missing. Responses including gay or lesbian, bisexual, pansexual, asexual, and other were collapsed into sexuality diverse due to small numbers.

Covariates were self-reported. Demographic variables included language spoken at home, household structure, perceived family socioeconomic status, and school location.

Individual and psychosocial variables included hyperactivity and peer problems assessed using the 5-item subscales (total score ranged from 0 to 10) from the Strengths and Difficulties Questionnaire (SDQ),^42^ with hyperactivity scores of 7 or higher indicating clinical-level symptoms (dichotomized as <7 and ≥7) and peer problems scores of 4 or higher indicating clinical-level symptoms (dichotomized as <4 and ≥4). Any physical or cognitive disability was dichotomized as yes (1 or more diagnoses) or no (none) from responses to whether the participant had autism or Asperger, intellectual disability, learning disability, Tourette syndrome, cerebral palsy, brain injury, neurological disability, hearing impairment, or visual impairment. Being bullied and alcohol or other drug use were dichotomized (yes or no). Maladaptive social media use was derived from the total score on the 7-item maladaptive Facebook use questionnaire, an adapted version,^43^ with total scores ranging from 7 to 49, and no social media use and was dichotomized as scores below the median indicating no or low maladaptive social media use and scores at the median or higher indicating high maladaptive social media use. Positive family support (2 items) and negative family interactions (3 items) were derived from the Schuster Social Support Scale,^44^ with higher scores on the supportive interactions scale indicating more supportive interactions and higher scores on the negative support indicating more negative interactions, and were dichotomized as low (scores below the median) or high (scores at the median or higher). School connectedness was assessed using the questionnaire developed by the Organisation for Economic Co-operation and Development (OECD) Programme for International Student Assessment (PISA),^45^ with the total score from 6 items scored as 1 (strongly disagree), 2 (disagree), 3 (agree), and 4 (strongly agree), scores ranging from 6 to 24 and higher scores indicating a greater sense of belonging. This assessment was dichotomized as scores below the median indicating low connectedness and scores at the median or higher indicating high connectedness. School climate (supportiveness) was also assessed using the OECD PISA,^45^ with total scores on 7 items scored as 0 (never), 1 (sometimes), and 2 (always), with scores ranging from 0 to 14. This assessment was dichotomized as scores below the median indicating less positive school climate and scores at or higher than the median indicating high positive school climate. Variable descriptions are presented in eTable 1 in Supplement 1.

### Statistical Analysis

All statistical analyses were conducted using the SAS procedure for survey data in SAS 9.4 (SAS Institute Inc). Intraclass correlation coefficients indicated that 5.2% (depression) and 6.0% (anxiety) of the variance was due to between-school differences; therefore, all analyses were adjusted for clustering by school to reduce bias. For bivariate analysis, Rao-Scott χ^2^ tests (a design-adjusted version of Pearson χ^2^ test) were used. Although outcome variables had 3 ordered categories, proportional odds assumptions for ordered logistic regression tests were violated for both outcomes. Therefore, multinomial logistic regressions were used for this study. Unadjusted and adjusted (for the aforementioned covariates) multinomial logistic regressions were performed to examine the associations between gender diversity and sexuality diversity and mental health symptoms.

Z tests were conducted for the equality of 2 proportions. Linear hypothesis tests were used to test the equality of 2 regression coefficients of GSD variables. All hypothesis tests were 2-sided, with statistical significance defined as *P* < .05. Odds ratios (ORs) and adjusted odds ratios (AOR) are presented with 95% CIs.

Missing data in GSD variables were modeled as a separate category using the SAS SURVEYLOGISTIC procedure, which allowed for estimation of mental health risks among adolescents who might be reluctant to disclose their gender or sexuality but reported depression or anxiety and retained all participants in the model, reducing bias. For missing data in all other covariates, multiple imputation (20 datasets) was used. Since SURVEYLOGISTIC did not handle missing data in outcomes and missingness was minimal (depression: n = 1; anxiety: n = 14), listwise deletion was used, with potential bias likely negligible. Sensitivity analyses conducted by excluding some potential mediators (interpersonal, family, and school factors) from the multivariable models examined the changes in the associations between GSD variables and outcomes.

## Results

### Demographic Characteristics of the Sample

Among 7577 written consent forms obtained, 6388 participants agreed to join the study (mean [SD] age, 13.9 [0.5] years), with 5842 (91.5%) born in Australia, 5982 (93.7%) spoke English at home, 5009 (78.4%) were from a 2-parent home, and 4949 (77.5%) reported medium or high perceived socioeconomic status (eTable 2 in Supplement 1). Sex at birth included 3329 (52.1%) female, 968 (46.5%) male, 33 (0.5%) unsure, 52 (0.8%) prefer not to say, and 6 (0.1%) another. Of the sample, 6019 (94.2%) reported a gender congruent with their sex at birth (cisgender), 209 (3.3%) reported a gender different from their sex at birth (gender diverse), 109 (1.7%) preferred not to report gender, and 51 (0.8%) were missing gender. Sexual orientation revealed 4472 (70.0%) were heterosexual, 767 (12.0%) were sexuality diverse, 559 (8.8%) were unsure, 296 (4.6%) preferred not to report, and 294 (4.6%) were missing. Detailed further disaggregated subidentities were published elsewhere.^5^ Briefly, subidentities included: 33 (0.5%) transgender, 117 (1.8%) nonbinary, and 59 (0.9%) other gender identities; 103 (1.6%) gay or lesbian, 414 (6.5%) bisexual, 119 (1.9%) pansexual, 67 (1.0%) asexual, and 64 (1.0%) other sexual identities. There were 162 participants (ie, 77.5% of the gender-diverse group and 21.1% of the sexuality-diverse group) who were both gender diverse and sexuality diverse. Descriptive statistics are presented in eTable 2 in Supplement 1.

### Bivariate Analysis of Sample Characteristics

Bivariate analysis (χ^2^ tests) of the GSD group with depression, anxiety, and covariates are presented in Table 1 (and eTable 2 in Supplement 1). In the overall sample, 3745 adolescents (58.6%) reported depression (2780 [43.5%] mild to moderate, 965 [15.1%] clinical level), and 1888 adolescents (29.6%) reported anxiety (958 [15.0%] elevated, 930 [14.6%] clinical level). Most covariates had associations with depression and anxiety symptoms (eTable 2 in Supplement 1).

**Table 1.** Characteristics of Study Sample by Gender and Sexuality Diversity (N = 6388)

Outcomes, demographics, and psychosocial characteristics	Sample total, No. (%)	Gender diversity, No. (%)				*P* value^a^	Sexuality diversity, No. (%)					*P* value^a^
		Cisgender	Gender-diverse	Prefer not to say	Missing		Heterosexual	Sexuality-diverse	Unsure	Prefer not to say	Missing	
**Outcomes**												
Depression												
No symptoms	2642 (41.4)	2575 (42.8)	22 (10.5)	20 (18.3)	25 (49.0)	<.001	2084 (46.6)	107 (14.0)	214 (38.3)	121 (40.9)	116 (39.5)	<.001
Mild to moderate	2780 (43.5)	2641 (43.9)	63 (30.1)	55 (50.5)	21 (41.2)		1934 (43.3)	327 (42.6)	258 (46.2)	122 (41.2)	139 (47.3)	
Clinical level	965 (15.1)	802 (13.3)	124 (59.3)	34 (31.2)	5 (9.8)		453 (10.1)	333 (43.4)	87 (15.6)	53 (17.9)	39 (13.3)	
Anxiety												
No symptoms	4486 (70.4)	4345 (72.3)	62 (29.7)	37 (34.3)	42 (84.0)	<.001	3406 (76.2)	302 (39.4)	374 (66.9)	195 (65.9)	209 (74.6)	<.001
Mild to moderate	958 (15.0)	874 (14.5)	45 (21.5)	37 (34.3)	2 (4.0)		579 (13.0)	194 (25.3)	95 (17.0)	49 (16.6)	41 (14.6)	
Clinical level	930 (14.6)	788 (13.1)	102 (48.8)	34 (31.5)	6 (12.0)		487 (10.9)	271 (35.3)	90 (16.1)	52 (17.6)	30 (10.7)	
**Covariates**												
Language spoken at home												
Other	405 (6.3)	373 (6.2)	17 (8.1)	10 (9.2)	5 (9.8)	.43	263 (5.9)	50 (6.5)	57 (10.2)	17 (5.7)	18 (6.1)	.02
English	5982 (93.7)	5645 (93.8)	192 (91.9)	99 (90.8)	46 (90.2)		4208 (94.1)	717 (93.5)	502 (89.8)	279 (94.3)	276 (93.9)	
Location												
Regional	1533 (24.0)	1446 (24.0)	59 (28.2)	16 (14.7)	12 (23.5)	.14	1077 (24.1)	173 (22.6)	127 (22.7)	86 (29.1)	70 (23.8)	.70
City	4855 (76.0)	4573 (76.0)	150 (71.8)	93 (85.3)	39 (76.5)		3395 (75.9)	594 (77.4)	432 (77.3)	210 (70.9)	224 (76.2)	
Socioeconomic status												
Low	529 (8.3)	491 (8.2)	24 (11.5)	7 (6.4)	7 (13.7)	.003	359 (8.0)	70 (9.1)	41 (7.3)	21 (7.1)	38 (12.9)	<.001
Medium or high	4949 (77.5)	4693 (78.0)	149 (71.3)	74 (67.9)	33 (64.7)		3504 (78.4)	600 (78.2)	443 (79.2)	199 (67.2)	203 (69.0)	
Prefer not to say	910 (14.2)	835 (13.9)	36 (17.2)	28 (25.7)	11 (21.6)		609 (13.6)	97 (12.6)	75 (13.4)	76 (25.7)	53 (18.0)	
Household structure												
Single-parent or other	1379 (21.6)	1263 (21.0)	67 (32.1)	33 (30.3)	16 (31.4)	<.001	890 (19.9)	204 (26.6)	130 (23.3)	77 (26.0)	78 (26.5)	<.001
2-Parent	5009 (78.4)	4756 (79.0)	142 (67.9)	76 (69.7)	35 (68.6)		3582 (80.1)	563 (73.4)	429 (76.7)	219 (74.0)	216 (73.5)	
Hyperactivity^b^												
Clinical (score ≥7)	1655 (26.3)	1461 (24.6)	127 (61.7)	56 (52.3)	11 (22.0)	<.001	1008 (22.6)	390 (50.8)	124 (22.2)	73 (24.7)	60 (28.4)	<.001
Nonclinical (score <7)	4648 (73.7)	4479 (75.4)	79 (38.3)	51 (47.7)	39 (78.0)		3462 (77.4)	377 (49.2)	435 (77.8)	223 (75.3)	151 (71.6)	
Peer problem^b^												
Clinical (score ≥4)	1539 (24.4)	1369 (23.0)	115 (55.8)	43 (40.2)	12 (24.0)	<.001	915 (20.5)	337 (43.9)	149 (26.7)	88 (29.7)	50 (23.7)	<.001
Nonclinical (score <4)	4764 (75.6)	4571 (77.0)	91 (44.2)	64 (59.8)	38 (76.0)		3555 (79.5)	430 (56.1)	410 (73.6)	208 (70.3)	161 (76.3)	
Being bullied												
Yes	2361 (38.0)	2176 (37.1)	120 (59.1)	43 (40.6)	22 (45.8)	<.001	1627 (36.4)	375 (48.9)	208 (37.2)	108 (36.5)	43 (33.9)	<.001
No	3860 (62.0)	3688 (62.9)	83 (40.9)	63 (59.4)	26 (54.2)		2845 (63.6)	392 (51.1)	351 (62.8)	188 (63.5)	84 (66.1)	
Physical or cognitive disability												
Yes	797 (12.5)	716 (11.9)	51 (24.4)	19 (17.4)	11 (21.6)	<.001	499 (11.2)	152 (19.8)	65 (11.6)	36 (12.2)	45 (15.3)	<.001
No	5591 (87.5)	5303 (88.1)	158 (75.6)	90 (82.6)	40 (78.4)		3973 (88.8)	615 (80.2)	494 (88.4)	260 (87.8)	249 (84.7)	
Alcohol or drug use												
Yes	1116 (18.1)	1026 (17.7)	56 (27.6)	23 (21.9)	11 (22.9)	.007	804 (18.0)	178 (23.2)	72 (12.9)	48 (16.2)	14 (20.9)	<.001
No	5045 (81.9)	4779 (82.3)	147 (72.4)	82 (78.1)	37 (77.1)		3668 (82.0)	589 (76.8)	487 (87.1)	248 (83.8)	53 (79.1)	
Positive support, family^c^												
Low	2565 (41.4)	2326 (39.9)	145 (71.4)	71 (67.6)	23 (47.9)	<.001	1616 (36.1)	486 (63.4)	277 (49.6)	147 (49.7)	39 (39.8)	<.001
High	3627 (58.6)	3510 (60.1)	58 (28.6)	34 (32.4)	25 (52.1)		2856 (63.9)	281 (36.6)	282 (50.4)	149 (50.3)	59 (60.2)	
Negative interaction, family^c^												
Low	3044 (49.2)	2936 (50.3)	48 (23.6)	32 (30.5)	28 (58.3)	<.001	2338 (52.3)	242 (31.6)	269 (48.1)	142 (48.0)	53 (54.1)	<.001
High	3148 (50.8)	2900 (49.7)	155 (76.4)	73 (69.5)	20 (41.7)		2134 (47.7)	525 (68.4)	290 (51.9)	154 (52.0)	45 (45.9)	
Maladaptive social media use^c^												
No or less maladaptive use	3220 (51.9)	3048 (52.1)	100 (49.3)	47 (44.3)	25 (52.1)	.39	2361 (52.8)	369 (48.1)	287 (51.3)	149 (50.3)	54 (46.6)	.16
High maladaptive use	2990 (48.1)	2805 (47.9)	103 (50.7)	59 (55.7)	23 (47.9)		2111 (47.2)	398 (51.9)	272 (48.7)	147 (49.7)	62 (53.4)	
School connectedness												
Low	2632 (42.3)	2376 (40.5)	160 (78.8)	71 (67.0)	25 (51.0)	<.001	1632 (36.5)	525 (68.4)	270 (48.3)	156 (52.7)	49 (37.1)	<.001
High	3594 (57.7)	3492 (59.5)	43 (21.2)	35 (33.0)	24 (49.0)		2840 (63.5)	242 (31.6)	289 (51.7)	140 (47.3)	83 (62.9)	
Positive school climate												
Low	2898 (49.6)	2661 (48.4)	142 (70.6)	67 (68.4)	28 (68.3)	<.001	1964 (46.8)	474 (64.1)	256 (50.4)	139 (49.1)	65 (56.5)	<.001
High	2945 (50.4)	2842 (51.6)	59 (29.4)	31 (31.6)	13 (31.7)		2233 (53.2)	266 (35.9)	252 (49.6)	144 (50.9)	50 (43.5)	

^a^ *P* value is from the Rao-Scott χ^2^ test.

^b^ Hyperactivity and peer problems assessed using the 5-item subscales (total score ranged from 0 to 10) from the Strengths and Difficulties Questionnaire, with hyperactivity scores of 7 or higher and peer problems scores of 4 or higher indicating clinical-level symptoms.

^c^ Family support variables (positive and negative support) and maladaptive social media use were categorized as below the median for low scores and at the median or higher for high scores; no social media use and less maladaptive social media use were combined in a single category.

Larger proportions of GSD adolescents reported clinical levels of depression (gender-diverse: 59.3% vs 13.3%, *P* < .001; sexuality-diverse: 43.4% vs 10.1%, *P* < .001), and anxiety (gender-diverse: 48.8% vs 13.1%, *P* < .001; sexuality-diverse: 35.3% vs 10.9%, *P* < .001) than cisgender and heterosexual peers, respectively (Table 1). Similarly, larger proportions of those preferring not to report gender or their sexuality also reported clinical levels of depression and anxiety. Larger proportions of gender-diverse and sexuality-diverse adolescents than their cisgender and heterosexual peers experienced clinical levels of hyperactivity and peer problems, bullying, disability, alcohol or other drugs use, lower levels of positive family support, higher levels of negative family interaction, lower school connectedness, and lower levels of positive school climate (Table 1). Similarly, larger proportions of adolescents preferring not to report gender or sexuality or were unsure about their sexuality, compared with reference groups, reported such adverse psychosocial characteristics. Most covariates showed bivariate associations with depression and anxiety symptoms (eTable 2 in Supplement 1) as well as GSD variables (Table 1), indicating these covariates as possible confounders.

### Associations of GSD Status With Depression and Anxiety

All 4 univariable logistic regressions (Table 2) showed that gender-diverse adolescents and sexuality-diverse adolescents had substantially higher odds of clinical levels (vs no symptoms) of depression (gender-diverse OR, 18.10 [95% CI, 11.48-28.52]; sexuality-diverse OR, 14.32 [95% CI, 11.16-18.36]) and anxiety (gender-diverse OR, 9.07 [95% CI, 6.77-12.15]; sexuality-diverse OR, 6.28 [95% CI, 5.07-7.77]), relative to their cisgender and heterosexual counterparts, respectively. Similarly, adolescents preferring not to report gender had higher odds of clinical levels (vs no symptoms) of depression (OR, 5.46 [95% CI, 3.14-9.49]) and anxiety (OR, 5.07 [95% CI, 3.14-8.17]), and those preferring not to report and unsure about their sexuality also had increased odds of clinical levels of depression (preferring not to report: OR, 2.02 [95% CI, 1.33-3.05]; unsure: OR, 1.87 [95% CI, 1.40-2.50]) and anxiety (preferring not to report: OR, 1.87 [95% CI, 1.31-2.66]; unsure: OR, 1.68 [95% CI, 1.28-2.21]).

**Table 2.** Univariable Logistic Regressions of Mental Health Outcomes by Gender Diversity and Sexuality Diversity

Symptom level (vs no symptoms), by identity group	OR (95% CI)			
	Depression		Anxiety	
	Model 1 (using gender diversity)^a^	Model 2 (using sexuality diversity)^a^	Model 3 (using gender diversity) ^a^	Model 4 (using sexuality diversity)^a^
**Gender diversity (vs cisgender)** ^b^				
Gender diverse				
Mild to moderate	2.79 (1.65-4.72)	NA	3.61 (2.53-5.15)	NA
Clinical level	18.10 (11.48-28.52)	NA	9.07 (6.77-12.15)	NA
Prefer not to report				
Mild to moderate	2.68 (1.55-4.63)	NA	4.97 (3.06-8.08)	NA
Clinical level	5.46 (3.14-9.49)	NA	5.07 (3.14-8.17)	NA
Missing				
Mild to moderate	0.82 (0.43-1.58)	NA	0.24 (0.05-1.08)	NA
Clinical level	0.64 (0.22-1.90)	NA	0.79 (0.31-1.98)	NA
**Sexuality diversity (vs heterosexual)**				
Sexuality diverse				
Mild to moderate	NA	3.29 (2.59-4.18)	NA	3.78 (3.05-4.69)
Clinical level	NA	14.32 (11.16-18.36)	NA	6.28 (5.07-7.77)
Unsure				
Mild to moderate	NA	1.30 (1.03-1.65)	NA	1.49 (1.12-1.99)
Clinical level	NA	1.87 (1.40-2.50)	NA	1.68 (1.28-2.21)
Prefer not to report				
Mild to moderate	NA	1.09 (0.84-1.40)	NA	1.48 (1.03-2.13)
Clinical level	NA	2.02 (1.33-3.05)	NA	1.87 (1.31-2.66)
Missing				
Mild to moderate	NA	1.29 (0.97-1.71)	NA	1.15 (0.79-1.69)
Clinical level	NA	1.55 (0.98-2.44)	NA	1.00 (0.65-1.55)

Abbreviations: NA, not applicable; OR, odds ratio.

^a^ Model 1 and model 3 included gender diversity and model 2 and model 4 included sexuality diversity as an independent variable.

^b^ Unsure category for gender diversity was not measured.

After adjusting for demographic, psychosocial, family, and school variables, all 4 logistic regressions (Table 3) still showed associations, although with reduced ORs, reflecting the confounding or explanatory roles of these covariates. In adjusted models, gender-diverse adolescents had higher odds of clinical levels (vs no symptoms) of depression (AOR, 5.68 [95% CI, 3.46-9.33]) and anxiety (AOR, 3.49 [95% CI, 2.46-4.95]) compared with cisgender adolescents. Sexuality-diverse adolescents had higher odds of clinical levels of depression (AOR, 6.69 [95% CI, 4.66-9.03]) and anxiety (AOR, 3.07 [95% CI, 2.40-3.93]) compared with their heterosexual counterparts. Adolescents preferring not to report gender had increased odds of clinical levels of anxiety (AOR, 2.88 [95% CI, 1.81-4.58]), and those who were unsure about their sexuality had higher clinical levels of depression (AOR, 1.67 [95% CI, 1.22-2.27]) and anxiety (AOR, 1.50 [95% CI, 1.11-2.03]). Furthermore, odds of mild to moderate levels of depression or anxiety were higher for gender-diverse adolescents (anxiety AOR, 1.88 [95% CI, 1.30-2.72]) and sexuality-diverse adolescents (depression AOR, 2.14 [95% CI, 1.63-2.81; anxiety AOR, 2.32 [95% CI, 1.86-2.89]). However, the odds were significantly higher for clinical levels of depression and anxiety than for mild or moderate levels among both gender-diverse adolescents and sexuality-diverse adolescents as shown by the linear hypothesis tests (Table 3).

**Table 3.** Multivariable Multinomial Logistic Regression Analyses of Depression and Anxiety Outcomes

Symptom level (vs no symptoms), by identity group and covariates	OR (95% CI)			
	Depression		Anxiety	
	Model 1 (using gender diversity)^a^	Model 2 (using sexuality diversity)^a^	Model 3 (using gender diversity)^a^	Model 4 (using sexuality diversity)^a^
**Gender diversity (vs cisgender)^b^**				
Gender diverse				
Mild to moderate	1.52 (0.94-2.47)	NA	1.88 (1.30-2.72)	NA
Clinical level	5.68 (3.46-9.33)	NA	3.49 (2.46-4.95)	NA
Prefer not to say				
Mild to moderate	1.37 (0.73-2.60)	NA	3.18 (1.95-5.19)	NA
Clinical level	1.74 (0.89-3.38)	NA	2.88 (1.81-4.58)	NA
Missing				
Mild to moderate	0.62 (0.29-1.31)	NA	0.18 (0.04-0.77)	NA
Clinical level	0.36 (0.09-1.41)	NA	0.56 (0.20-1.60)	NA
**Sexuality diversity (vs heterosexual)**				
Sexuality diverse				
Mild to moderate	NA	2.14 (1.63-2.81)	NA	2.32 (1.86-2.89)
Clinical level	NA	6.49 (4.66-9.03)	NA	3.07 (2.40-3.93)
Unsure				
Mild to moderate	NA	1.18 (0.92-1.51)	NA	1.34 (1.01-1.78)
Clinical level	NA	1.67 (1.22-2.27)	NA	1.50 (1.11-2.03)
Prefer not to say				
Mild to moderate	NA	0.94 (0.72-1.23)	NA	1.31 (0.91-1.87)
Clinical level	NA	1.57 (0.98-2.52)	NA	1.55 (1.05-2.30)
Missing				
Mild to moderate	NA	1.16 (0.83-1.62)	NA	0.98 (0.67-1.42)
Clinical level	NA	1.19 (0.67-2.21)	NA	0.74 (0.47-1.16)
**Covariates**				
Hyperactivity: clinical (vs nonclinical)				
Mild to moderate	3.54 (2.94-4.25)	3.47 (2.88-4.18)	2.17 (1.82-2.59)	2.12 (1.77-2.53)
Clinical level	11.71 (9.31-14.71)	11.18 (8.85-14.13)	3.86 (3.19-4.68)	3.78 (3.13-4.56)
Peer problem: clinical (vs nonclinical)				
Mild to moderate	1.37 (1.15-1.63)	1.36 (1.14-1.63)	1.49 (1.22-1.81)	1.46 (1.19-1.78)
Clinical level	2.63 (2.11-3.29)	2.53 (2.03-3.14)	2.21 (1.81-2.70)	2.16 (1.77-2.64)
Being bullied: yes (vs no)				
Mild to moderate	1.40 (1.21-1.62)	1.40 (1.21-1.62)	1.39 (1.19-1.64)	1.41 (1.20-1.65)
Clinical level	1.60 (1.28-2.00)	1.67 (1.34-2.08)	1.82 (1.52-2.19)	1.87 (1.56-2.25)
Physical or cognitive disability: yes (vs no)				
Mild to moderate	1.59 (1.30-1.94)	1.54 (1.25-1.88)	1.24 (1.00-1.53)	1.19 (0.96-1.48)
Clinical level	1.71 (1.29-2.26)	1.63 (1.24-2.15)	1.24 (0.96-1.60)	1.22 (0.95-1.58)
Alcohol or drug use: yes (vs no)				
Mild to moderate	1.40 (1.15-1.71)	1.43 (1.17-1.74)	0.91 (0.72-1.14)	0.92 (0.73-1.16)
Clinical level	1.88 (1.47-2.41)	1.98 (1.55-2.53)	0.99 (0.78-1.26)	1.01 (0.79-1.27)
Positive family support: high (vs low)^c^				
Mild to moderate	0.61 (0.52-0.72)	0.63 (0.53-0.73)	0.82 (0.70-0.95)	0.85 (0.73-0.99)
Clinical level	0.36 (0.29-0.44)	0.38 (0.31-0.48)	0.76 (0.63-0.92)	0.80 (0.66-97)
Negative family interaction: high (vs low)^c^				
Mild to moderate	2.02 (1.77-2.30)	2.02 (1.78-2.30)	1.68 (1.40-2.00)	1.67 (1.40-2.00)
Clinical level	3.18 (2.54-3.99)	3.22 (2.56-4.05)	1.92 (1.56-2.36)	1.94 (1.58-2.37)
Social media maladaptive use: high (vs low)^c^				
Mild to moderate	1.25 (1.09-1.42)	1.26 (1.10-1.44)	1.30 (1.11-1.52)	1.32 (1.12-1.55)
Clinical level	1.53 (1.23-1.90)	1.55 (1.25-1.93)	1.66 (1.41-1.96)	1.67 (1.41-1.98)
School connectedness: low (vs high)^c^				
Mild to moderate	2.25 (1.92-2.65)	2.19 (1.86-2.58)	2.49 (2.04-3.04)	2.39 (1.96-2.92)
Clinical level	4.07 (3.16-5.23)	3.76 (2.93-4.83)	3.80 (2.97-4.85)	3.62 (2.85-4.61)
Positive school climate: low (vs high)^c^				
Mild to moderate	1.39 (1.21-1.60)	1.39 (1.21-1.59)	1.26 (1.06-1.49)	1.27 (1.07-1.50)
Clinical level	1.77 (1.41-2.23)	1.82 (1.44-2.28)	1.01 (0.82-1.26)	1.04 (0.83-1.29)
Perceived socioeconomic status: low (vs medium or high)				
Mild to moderate	1.19 (0.93-1.51)	1.19 (0.94-1.52)	1.13 (0.87-1.46)	1.15 (0.89-1.49)
Clinical level	1.38 (1.05-1.82)	1.46 (1.09-1.94)	1.33 (1.03-1.73)	1.38 (1.07-1.79)
School location: major cities (vs regional)				
Mild to moderate	1.42 (1.17-1.72)	1.40 (1.16-1.70)	1.17 (0.98-1.39)	1.16 (0.98-1.38)
Clinical level	1.49 (1.12-1.98)	1.42 (1.09-1.85)	1.17 (0.94-1.44)	1.14 (0.93-1.41)
Language spoken at home: other (vs English)				
Mild to moderate	1.26 (0.99-1.60)	1.26 (0.99-1.61)	1.24 (0.95-1.62)	1.23 (0.94-1.62)
Clinical level	1.40 (0.99-1.97)	1.40 (0.96-2.04)	0.80 (0.58-1.11)	0.81 (0.57-1.13)
Single parent, blended, or other (vs 2 parents)				
Mild to moderate	1.02 (0.86-1.21)	1.02 (0.86-1.20)	0.90 (0.74-1.10)	0.90 (0.74-1.09)
Clinical level	1.22 (0.95-1.55)	1.21 (0.95-1.53)	1.05 (0.85-1.31)	1.05 (0.85-1.30)

Abbreviations: NA, not applicable; OR, odds ratio.

^a^ Model 1 and model 3 included gender diversity and model 2 and model 4 included sexuality diversity as an independent variable.

^b^ Unsure category for gender diversity was not measured.

^c^ High refers to scores at the median or higher, and low refers to scores below the median.

For the sample, several covariates in all 4 models (Table 3) were associated with both mental health symptoms. The odds of clinical levels and mild to moderate symptoms of depression and anxiety (vs no symptoms) were significantly higher for adolescents who reported higher levels of hyperactivity and peer problems, being bullied, higher negative family interactions, higher maladaptive social media use, and lower school connectedness. Conversely, adolescents having higher positive family support had reduced odds of both mild to moderate and clinical levels of depression and anxiety.

Sensitivity analyses excluding potential mediating factors (hyperactivity, peer problems, being bullied, positive and negative family support, and school climate) revealed higher odds for associations with GSD status, mainly gender-diverse and sexuality-diverse, and depression and anxiety (Table 4). When these variables were included (Table 3), the odds for the associations were attenuated, suggesting that these factors may partly explain the elevated depression and anxiety among GSD adolescents, although causal mediation cannot be inferred from cross-sectional data.

**Table 4.** Multivariable Multinomial Logistic Regressions of Depression and Anxiety Outcomes by Excluding Potential Mediating Variables^a^

Symptom level (vs no symptoms), by identity group and covariates	OR (95% CI)			
	Depression		Anxiety	
	Model 1 (using gender diversity)^b^	Model 2 (using sexuality diversity)^b^	Model 3 (using gender diversity)^b^	Model 4 (using sexuality diversity)^b^
**Gender diversity (vs cisgender)^c^**				
Gender diverse				
Mild to moderate	2.05 (1.22-3.43)	NA	2.57 (1.82-3.61)	NA
Clinical level	10.78 (6.85-16.97)	NA	5.71 (4.19-7.78)	NA
Prefer not to say				
Mild to moderate	1.97(1.15-3.37)	NA	3.99 (2.46-6.48)	NA
Clinical level	3.44 (1.99-5.93)	NA	3.86 (2.43-6.14)	NA
Missing				
Mild to moderate	0.60 (0.31-1.17)	NA	0.18 (0.04-0.74)	NA
Clinical level	0.35 (0.11-1.13)	NA	0.53 (0.20-1.36)	NA
**Sexuality diversity (vs heterosexual)**				
Sexuality diverse				
Mild to moderate	NA	2.69 (2.13-3.40)	NA	2.88 (2.35-3.53)
Clinical level	NA	10.20 (7.95-13.09)	NA	4.30 (3.46-5.33)
Unsure				
Mild to moderate	NA	1.21 (0.94-1.54)	NA	1.33 (1.01-1.76)
Clinical level	NA	1.66 (1.22-2.24)	NA	1.46 (1.10-1.94)
Prefer not to say				
Mild to moderate	NA	0.93 (0.73-1.20)	NA	1.26 (0.88-1.79)
Clinical level	NA	1.55 (1.01-2.36)	NA	1.47 (1.02-2.12)
Missing				
Mild to moderate	NA	1.20 (0.89-1.60)	NA	1.04 (0.72-1.51)
Clinical level	NA	1.32 (0.80-2.18)	NA	0.84 (0.55-1.29)
**Covariates**				
Physical or cognitive disability: yes (vs no)				
Mild to moderate	1.75 (1.45-2.11)	1.70 (1.41-2.06)	1.39 (1.13-1.72)	1.34 (1.09-1.66)
Clinical level	2.08 (1.61-2.70)	1.99 (1.55-2.55)	1.54 (1.21-1.96)	1.50 (1.18-1.90)
Alcohol or drug use: yes (vs no)				
Mild to moderate	1.95 (1.64-2.33)	2.00 (1.68-2.38)	1.23 (0.99-1.52)	1.24 (1.00-1.54)
Clinical level	3.50 (2.83-4.32)	3.68 (2.99-4.54)	1.52 (1.22-1.89)	1.53 (1.23-1.90)
Social media maladaptive use: high (vs low)^d^				
Mild to moderate	1.42 (1.26-1.61)	1.44 (1.27-1.62)	1.43 (1.23-1.67)	1.45 (1.24-1.69)
Clinical level	1.91 (1.59-1.30)	1.94 (1.61-2.34)	1.91 (1.63-2.23)	1.92 (1.63-2.26)
School connectedness: low (vs high)^d^				
Mild to moderate	3.62 (3.14-4.18)	3.51 (3.04-4.06)	3.92 (3.29-4.67)	3.68 (3.09-4.39)
Clinical level	11.16 (8.85-14.09)	10.04 (8.01-12.60)	7.68 (6.16-9.58)	7.13 (5.75-8.84)
Perceived socioeconomic status: low (vs medium or high				
Mild to moderate	1.30 (1.02-1.66)	1.33 (1.04-1.70)	1.22 (0.95-1.58)	1.23 (0.95-1.60)
Clinical level	1.64 (1.27-2.11)	1.71 (1.30-2.25)	1.49 (1.17-1.90)	1.52 (1.19-1.95)
School location: major cities (vs regional)				
Mild to moderate	1.37 (1.13-1.66)	1.36 (1.13-1.64)	1.16 (0.97-1.37)	1.15 (0.98-1.35)
Clinical level	1.39 (1.05-1.84)	1.32 (1.03-1.71)	1.10 (0.90-1.35)	1.07 (0.88-1.30)
Language spoken at home: other (vs English				
Mild to moderate	1.31 (1.03-1.67)	1.30 (1.02-1.66)	1.24 (0.96-1.61)	1.23 (0.95-1.60)
Clinical level	1.31 (0.92-1.89)	1.34 (0.91-1.97)	0.76 (0.55-1.06)	0.77 (0.54-1.09)
Single parent, blended, or other (vs 2-parents)				
Mild to moderate	1.08 (0.93-1.25)	1.06 (0.92-1.23)	0.95 (0.79-1.15)	0.95 (0.79-1.14)
Clinical level	1.40 (1.14-1.72)	1.40 (1.14-1.71)	1.17 (0.96-1.43)	1.17 (0.96-1.43)

Abbreviations: NA, not applicable; OR, odds ratio

^a^ These analyses excluded hyperactivity, peer problems, being bullied, positive family support, negative family interaction, and positive school climate, which may lie on the path between GSD and mental health outcomes.

^b^ Model 1 and model 3 included gender diversity and model 2 and model 4 included sexuality diversity as an independent variable.

^c^ Unsure category for gender diversity was not measured.

^d^ High refers to scores at the median or higher, and low refers to scores below the median.

## Discussion

This cross-sectional study presents the prevalence rates and risks of depression and anxiety in younger adolescents, focusing on GSD in early adolescents (mean age, 13.9 years), using one of the largest, most comprehensive, and broadly representative datasets in this age group. We examined mental health disparities with disaggregated categories, including gender-diverse, sexuality-diverse, persons who preferred not to report their gender and sexuality, persons who were unsure about their sexuality, and persons missing reporting their gender or sexuality, compared with their cisgender and heterosexual counterparts. This disaggregation is an important distinction, less commonly explored in prior research, enabling us to present novel and meaningful evidence of varying risks of depression and anxiety for each category of these GSD groups. We found higher proportions of gender-diverse (3.3%) and sexuality-diverse (12.0%) adolescents than some recent school-based international studies, such as 1.4% gender diverse and 9.1% sexuality diverse among 9th through 11th graders in the US^2^ and 2.0% gender diverse among New Zealand students aged 12 to 18 years (school years, 9-13).^46^ Other US studies, however, reported higher prevalence rates, including 9.2% gender diverse among 9th through 12th graders^47^ and 22.2% sexuality diverse among 8th through 11th graders.^48^ These differences in prevalence rates likely reflect variations in survey response options, sample age groups, cultural context, degree of comfort in reporting, operationalization of gender identity, access to gender-affirming care, and secular shifts in sexual orientation identity.^47,48^ Surveys with broader response options generally reported higher prevalence rates.^48^ Given these factors, and wider age ranges (grades 8 through 12) in prior studies, direct comparisons across findings are difficult. Importantly, despite adolescents’ overall elevated risk of mental health problems^24,49,50^ and the relatively small GSD subpopulation, depression and anxiety were far more common among GSD adolescents in the present study, with 59.3% of gender-diverse (vs 13.3% of cisgender) adolescents and 43.4% of sexuality-diverse (vs 10.1% of heterosexual) adolescents reporting clinical levels of depression, and 48.8% of gender-diverse (vs 13.1% of cisgender) adolescents and 35.3% of sexuality-diverse (vs 10.9% of heterosexual) adolescents reporting clinical levels of anxiety. Our findings align with Australian online self-selected surveys of sexuality- and gender-diverse youths,^51,52,53^ showing that 61% of LGBTQ+ youths and 40% of same-sex–attracted adolescents (aged 14-21 years) experienced high or very high levels of psychological distress, with gender-diverse youths showing elevated rates,^51^ and 3 in 4 trans young people (aged 14-25 years) having depression, anxiety, or both.^53^ Additionally, larger proportions of adolescents in our study who preferred not to report gender and sexuality and who were unsure about their sexuality reported greater symptoms of depression and anxiety than their cisgender and heterosexual peers. While individuals indicating that they preferred not to report and were unsure may include some cisgender or heterosexual adolescents, many are possibly still navigating their gender or sexuality identities and face stigma, bullying, and discrimination,^5,54^ which can contribute to poorer mental health.

Multivariable analyses in the present study showed that gender-diverse adolescents had 5-fold and 3-fold higher odds, and sexuality-diverse adolescents had 6-fold and 3-fold higher odds, of clinical levels of depression and anxiety, respectively, compared with their cisgender and heterosexual peers. These findings align with prior research showing greater mental health risks for GSD groups,^13,16,18,53,55^ including 1 study reporting 5-fold greater odds of high levels of depressive symptoms for sexual minorities.^13^ Direct comparisons across studies remain difficult given variations in symptom measures, cutoffs, sample characteristics, and, importantly, how gender and sexuality diversity are defined and grouped. Previous research often aggregates gender and sexuality diversity under the LGBTQA+ umbrella or focuses on a single identity,^13,16,18,53,55^ overlooking subgroup differences in mental health risks, as evidenced by our findings.

The findings of elevated odds of clinical levels of anxiety among adolescents preferring not to report gender identity, and higher odds of clinical levels of depression and anxiety among adolescents unsure about their sexuality, emphasize the importance of examining risks across extended heterogeneous groups. Higher anxiety among individuals preferring not to report gender may reflect stigma, bullying, discrimination, or fear of disclosure,^5^ and adolescents unsure of their gender or sexuality face adverse outcomes, including substance use,^56^ victimization, and poor mental health,^2,26,56^ which may explain the greater symptoms observed in our study. Although the small number prevented further disaggregation (eg, bisexual, pansexual, nonbinary, lesbian, gay, queer, and trans), our findings revealed different risks of depression and anxiety among gender and sexuality diversity categories, indicating varying interplay of risk and protective factors. More importantly, the higher prevalence rates and risks of mental health symptoms among GSD adolescents emphasize the need for immediate clinical treatments, tailored interventions, and preventive initiatives from early adolescence to reduce mental health inequities and unmet needs in these groups^27,30,57,58^ and to avert the trajectory of mental health problems.^59^

The findings also highlight the importance of accounting for multiple intersecting risk and protective factors. Univariable models showed sizable odds of clinical levels of depression (18-fold and 14-fold greater) and anxiety (9-fold and 6-fold greater) for gender-diverse adolescents and sexuality-diverse adolescents respectively, compared with their cisgender and heterosexual peers. After adjusting for all covariates, the odds decreased markedly yet remained significantly elevated, indicating confounding or mitigating roles of covariates. Some interpersonal and family factors may lie on the mediating or explanatory pathway linking GSD to mental health outcomes.^11^ Sensitivity analyses excluding hyperactivity, peer problems, being bullied, family support, and school climate showed substantially higher odds of associations between GSD and depression and anxiety, suggesting that these factors may partly explain the elevated risk of depression and anxiety observed among GSD adolescents. However, these findings should not be interpreted as evidence of mediation due to the cross-sectional design of the study. Nonetheless, these findings align with evidence that GSD adolescents face compounded risks from individual psychopathologies and inability to cope, environmental and social challenges, including family rejection, peer bullying, and homelessness, along with minority-related stressors (stigma, prejudice, and victimization), contributing to heightened mental health risks,^9,10,11,53,60,61,62^ while supportive family, school, and availability and accessibility of mental health support and gender-affirming care are more protective.^21,53,60,62,63^ These findings underscore that clinicians, health care professionals, educational providers, and communities must consider both risks and supports for prevention and interventions from early adolescence.^2^ Additionally, GSD adolescents in Australia, particularly gender-diverse adolescents, remain vulnerable to discrimination and homophobic or transphobic abuse.^64^ Evidence from the US shows that supportive school climates reduce bullying and harassment reporting among GSD adolescents,^60^ suggesting the need for universally delivered stigma reduction and bullying prevention programs, along with expanded inclusive mental health services for GSD adolescents.

### Strengths and Limitations

This study has strengths. It used a large, comprehensive Australian dataset, allowing for robust subgroup analyses of gender and sexuality minority groups, including categories of preferred not to say or unsure about gender or sexuality, often overlooked groups. Adjusting for multiple potential confounders strengthened the validity of our results by reducing spurious associations while clarifying those between gender and sexuality diversity and mental health in early adolescence.

However, study limitations include the cross-sectional, opt-in, nonrandomized design, which prevents causal inference. Small absolute numbers of GSD adolescents restricted interaction tests and finer subgroup comparisons and limited capture of gender diversity, as *unsure* was an option only for sex at birth, not current gender. Most gender-diverse adolescents were also sexuality diverse, but heterosexual gender-diverse youths were too few for a separate analysis of intersecting diversity. Assessment of cultural and linguistic diversity followed the Australian Bureau of Statistics standards,^65^ but Indigenous data could not be disaggregated, as this requires approval by the local Indigenous human research ethics committees and leadership from Indigenous communities.

## Conclusions

In this cross-sectional study of young adolescents, gender-diverse adolescents and sexuality-diverse adolescents had significantly higher odds of greater depression and anxiety symptoms than their cisgender and heterosexual peers, underscoring the urgent need for multifaceted mental health interventions and prevention from early adolescence. Strategies must address identity-specific stressors and broader risks through bullying prevention, family support, adaptive social media use, improved school climate and connectedness, and accessible health services. Future longitudinal research should evaluate the population-level effectiveness of such interventions.

## Notes

Supplement 1. **eTable 1.** Description of independent and some covariates (instruments) **eTable 2.** Distribution of sample across the levels of demographic and psychosocial characteristics, and bivariate association with outcomes (N=6388)

Supplement 2.
Data Sharing Statement
