# Supplementary material for: Depression and Anxiety Among Young Gender- and Sexuality-Diverse Adolescents
Source: JAMA Netw Open. 2025 Dec 29;8(12):e2551570. doi: 10.1001/jamanetworkopen.2025.51570 (PMC12750252; doi:10.1001/jamanetworkopen.2025.51570)
Supplement: Supplement 1. — eTable 1. Description of independent and some covariates (instruments) eTable 2. Distribution of sample across the levels of demographic and psychosocial characteristics, and bivariate association with outcomes (N=6388) [file jamanetwopen-e2551570-s001.pdf]

Supplementary Online Content

Bista S, Werner-Seidler A, Maston K, et al. Depression and anxiety among young gender- and sexuality-diverse adolescents *JAMA Netw Open*. 2025;8(12):e2551570.
doi:10.1001/jamanetworkopen.2025.51570

eTable 1. Description of independent and some covariates (instruments)

eTable 2. Distribution of sample across the levels of demographic and psychosocial characteristics, and bivariate association with outcomes (N=6388)

This supplementary material has been provided by the authors to give readers additional information about their work.

© 2025 Bista S et al. *JAMA Network Open*.

eTable 1. Description of independent and some covariates (instruments)

Question	Response options	Explanation of derived variable
What sex were you assigned at birth, on your original birth certificate?	Female<br>Male<br>Not sure<br>I prefer not to say<br>Another term (specify)	
What is your current gender identity?	Female<br>Male<br>Non-binary<br>Other (specify)<br>I prefer not to say	Current gender identity (or gender diversity) for the analysis was derived using responses to 'sex at birth' and 'current gender identity' questions, and categorized as: 'cisgender', 'gender-diverse', 'prefer not to say (PNTS)', and 'missing'.
Do you consider your sexual orientation to be	Heterosexual or straight<br>Gay or lesbian<br>Bisexual<br>Pansexual<br>Asexual<br>Other (specify)<br>Not sure<br>Prefer not to say	Sexuality diversity for the analysis was categorized as: 'heterosexual', 'sexuality-diverse', 'prefer not to say (PNTS)', 'not sure (NS)', and 'missing'.
**Strength and Difficulties Questionnaire (SDQ) - 25 items in total including other sub-scales**		
**Hyperactivity Aggregate score: 5 items**		Score range: 0-10; Clinical cut-off raw score for Hyperactivity subscale: scores ≥ 7.
I am restless, I cannot stay still for long (SDQ2)	0 (Note true), 1 (Somewhat true), 2 (Certainly true)	
I am constantly fidgeting or squirming (SDQ10)	0 (Note true), 1 (Somewhat true), 2 (Certainly true)	
I am easily distracted; I find it difficult to concentrate (SDQ15)	0 (Note true), 1 (Somewhat true), 2 (Certainly true)	
I think before I do things (SDQ21)	0 (Note true), 1 (Somewhat true), 2 (Certainly true)	
I finish the work I'm doing. My attention is good (SDQ25)	0 (Note true), 1 (Somewhat true), 2 (Certainly true)	
**Peer problems - 5 items**		Score range: 0-10; Clinical cut-off raw score for Peer Problems: scores ≥ 4.
I would rather be alone than with people of my age (SDQ6)	0 (Note true), 1 (Somewhat true), 2 (Certainly true)	
I have one good friend or more (SDQ11)	0 (Note true), 1 (Somewhat true), 2 (Certainly true)	
Other people my age generally like me (SDQ14)	0 (Note true), 1 (Somewhat true), 2 (Certainly true)	
Other children or young people pick on me or bully me (SDQ19)	0 (Note true), 1 (Somewhat true), 2 (Certainly true)	

© 2025 Bista S et al. *JAMA Network Open*.

I get along better with adults than with people my own age (SDQ23)	0 (Note true), 1 (Somewhat true), 2 (Certainly true)	
**Schuster Social Support Scale (SSSS) - 10 items in total including 'friends' sub-scales**		Score range = 1-4 for both sub-scales
**SSSS_Family_Positive**	*Note: Response values were recoded.*	Higher scores indicate more supportive family.
How often do family make you feel cared for? (SSSS6)	1 (Never), 2 (Rarely), 3 (Sometimes), 4 (Often)	Dichotomized: 'low positive support' (<Median score], 'high positive support' (>=Median score).
How often do family express interest in how you are doing? (SSSS7)	1 (Never), 2 (Rarely), 3 (Sometimes), 4 (Often)	
**SSSS_Family_Negative**	*Note: Response values were recoded.*	Higher scores indicate more negative support. Dichotomized: 'low negative support' (<Median score), 'high negative support' (>=Median score).
How often do they make too many demands on you? (SSSS8)	1 (Never), 2 (Rarely), 3 (Sometimes), 4 (Often)	
How often do family criticize you? (SSSS9)	1 (Never), 2 (Rarely), 3 (Sometimes), 4 (Often)	
How often do they create tensions or arguments with you? (SSSS10)	1 (Never), 2 (Rarely), 3 (Sometimes), 4 (Often)	
**Maladaptive Social Media Use (MFUQ-Adopted) - 7 items**	1 (Strongly disagree) 2 (Disagree), 3 (Disagree somewhat), 4 (Neither agree nor disagree), 5 (Agree somewhat), 6 (Agree), 7 (Strongly agree)	
When I update my social media status, I expect others to comment on it.		
When I update my social media status and no one comments on it, I tend to be disappointed.	The same response options for all items	
I tend to read the social media status updates of others to see if others are feeling the way I am.		
When I update my social media status, it does not affect me if no one comments on it.		Reverse score item 4, then sum items: 1-7
I update my social media status multiple times per day.		
Reading the social media status updates of others tends to make me feel down on myself.		
I sometimes write negative things about myself in my social media status updates to see if others will respond with negative comments about me.		
**School connectedness - 6 items**	*Note: Response values were recoded.*	Score range: 6-24.
I make friends easily at school (SCHC1)	1 (Strongly disagree), 2 (Disagree), 3 (Agree), 4 (Strongly agree)	
I feel like I belong at school (SCHC2)	1 (Strongly disagree), 2 (Disagree), 3 (Agree), 4 (Strongly agree)	
Other students seem to like me (SCHC3)	1 (Strongly disagree), 2 (Disagree), 3 (Agree), 4 (Strongly agree)	School Connectedness Total Score = sum of all items. Higher scores indicate a greater sense of belonging. Dichotomized with 'low connectedness' (<Median score) and 'high connectedness' (>=Median score).
I feel like an outsider (or left out of things) at school (SCHC4)	4 (Strongly disagree), 3 (Disagree), 2 (Agree), 1 (Strongly agree)	
I feel awkward and out of place in my school (SCHC5)	4 (Strongly disagree), 3 (Disagree), 2 (Agree), 1 (Strongly agree)	
I feel lonely at school (SCHC6)	4 (Strongly disagree), 3 (Disagree), 2 (Agree), 1 (Strongly agree)	
**School climate - 7 items**		

© 2025 Bista S et al. *JAMA Network Open*.

At this school we care about each other (CLIMATE1)	0 (Never), 1 (Sometimes), 2 (Always)	
At this school we like each other (CLIMATE2)	0 (Never), 1 (Sometimes), 2 (Always)	
We can talk to teachers about problems (CLIMATE3)	0 (Never), 1 (Sometimes), 2 (Always)	School Climate Total Score = sum of all items. Range = 0-14. Higher scores indicate a more positive school climate. Dichotomized: ‘low positive’ (<Median score), ‘high positive’ (>=Median score).
Teachers try hard to help us (CLIMATE4)	0 (Never), 1 (Sometimes), 2 (Always)	
We feel safe in school (CLIMATE5)	0 (Never), 1 (Sometimes), 2 (Always)	
Our teachers are fair (CLIMATE6)	0 (Never), 1 (Sometimes), 2 (Always)	
There is an adult in my school who understands how I feel (CLIMATE7)	0 (Never), 1 (Sometimes), 2 (Always)	

© 2025 Bista S et al. *JAMA Network Open*.

eTable 2. Distribution of sample across the levels of demographic and psychosocial characteristics, and bivariate association with outcomes (N=6388)

Demographic and Psychosocial Characteristics	Sample Total N (%)	Depression, N (%)				Anxiety, N (%)			
		No symptoms	Mild-Moderate	Clinical levels	P-value	No symptoms	Mild-Moderate	Clinical levels	P-value
Language spoken at home									
Other	405 (6.3)	145 (35.8)	196 (48.4)	64 (15.8)	0.09	283 (69.9)	76 (18.8)	46 (11.4)	0.02
English	5982 (93.7)	2496 (41.7)	2584 (43.2)	901 (15.1)		4202 (70.4)	882 (14.8)	884 (14.8)	
Born in Australia									
No	546 (8.5)	224 (41.0)	241 (44.1)	81 (14.8)	0.96	390 (71.4)	87 (15.9)	69 (12.6)	0.33
Yes	5842 (91.5)	2418 (41.4)	2539 (43.5)	884 (15.1)		4096 (70.3)	871 (14.9)	861 (14.8)	
Location									
Regional	1533 (24.0)	652 (42.5)	625 (40.8)	256 (16.7)	0.19	1045 (68.8)	224 (14.6)	253 (16.5)	0.18
City	4855 (76.0)	1990 (41.0)	2155 (44.4)	709 (14.6)		3432 (70.9)	734 (15.2)	677 (14.0)	
Socio-economic status									
Low	529 (8.3)	154 (29.1)	245 (46.3)	130 (24.6)	<.001	312 (59.0)	93 (17.6)	124 (23.4)	<.001
Med-high	4949 (77.5)	2100 (42.4)	2159 (43.6)	689 (13.9)		3534 (71.6)	732 (14,8)	672 (13.6)	
PNTS	910 (14.2)	388 (42.6)	376 (41.3)	146 (16.0)		640 (70.6)	133 (14.7)	134 (14.7)	
Household structure									
Single-parent/Blended/Other	1379 (21.6)	465 (33.7)	608 (44.1)	305 (22.1)	<.001	881 (64.1)	217 (15.8)	277 (20.1)	<.001
Two-parent	5009 (78.4)	2177 (43.5)	2172 (43.4)	660 (13.2)		3350 (72.1)	741 (14.8)	653 (13.1)	
Gender diversity									
Cisgender	6019 (94.2)	2575 (42.8)	2641 (43.9)	802 (13.3)	<.001	4345 (72.3)	874 (14.5)	788 (13.1)	<.001
Gender-diverse	209 (3.3)	22 (10.5)	63 (30.1)	124 (59.3)		62 (29.7)	45 (21.5)	102 (48.8)	
Prefer not to report	109 (1.7)	20 (18.3)	55 (50.5)	34 (31.2)		37 (34.3)	37 (34.3)	34 (31.5)	
Missing	51 (0.80)	25 (49.0)	21 (41.2)	5 (9.8)		42 (84.0)	2 (4.0)	6 (12.0)	
Sexuality diversity									
Heterosexual/straight	4472 (70.0)	2084 (46.6)	1934 (43.3)	453 (10.1)	<.001	3406 (76.2)	579 (13.0)	487 (10.9)	<.001
Sexuality-diverse	767 (12.0)	107 (14.0)	327 (42.6)	333 (43.4)		302 (39.4)	194 (25.3)	271 (35.3)	
Unsure	559 (8.8)	214 (38.3)	258 (46.2)	87 (15.6)		374(66.9)	95 (17.0)	90 (16.1)	
Prefer not to report	296 (4.6)	121 (40.9)	122 (41.2)	53 (17.9)		195 (65.9)	49 (16.6)	52 (17.6)	
Missing	294 (4.6)	116 (39.5)	139 (47.3)	39 (13.3)		209 (74.6)	41 (14.6)	30 (10.7)	
Hyperactivity									
Clinical (score>=7)	1655 (26.3)	196 (11.8)	825 (49.8)	634 (38.3)	<.001	734 (44.4)	380 (23.0)	541 (32.7)	<.001
Non-clinical (score<7)	4648 (73.7)	2413 (51.9)	1914 (41.2)	320 (6.9)		3695 (79.5)	569 (12.2)	384 (8.3)	
Peer-problem									
Clinical (score>=4)	1539 (24.4)	287 (18.6)	735 (47.8)	517 (33.6)	<.001	693 (45.0)	347 (22.5)	499 (32.4)	<.001
Non-clinical (score<4)	4764 (75.6)	2322 (48.8)	2004 (42.1)	437 (9.2)		3736 (78.4)	602 (12.6)	426 (8.9)	
Being bullied									

© 2025 Bista S et al. *JAMA Network Open.*

Yes	2361 (38.0)	659 (27.9)	1151 (48.8)	551 (23.3)	<.001	1328 (56.2)	461 (19.6)	572 (24.2)	<.001
No	3860 (62.0)	1919 (49.7)	1552 (40.2)	388 (10.1)		3042 (78.8)	476 (12.3)	342 (8.9)	
Physical/cognitive disability									
Yes	797 (12.5)	219 (27.5)	399 (50.1)	179 (22.5)	<.001	475 (59.8)	149 (18.8)	170 (21.4)	<.001
No	5591 (87.5)	2423 (43.3)	2381 (42.6)	786 (14.1)		4011 (71.9)	809 (14.5)	760 (13.6)	
Alcohol use									
Yes	860 (14.0)	198 (23.0)	427 (49.7)	235 (27.3)	<.001	520 (60.5)	148 (17.2)	192 (22.3)	<.001
No	5301 (86.0)	2350 (44.3)	2248 (42.4)	702 (13.2)		3804 (71.7)	779 (14.5)	718 (13.5)	
Other substance use									
Yes	530 (8.6)	107 (20.2)	223 (42.1)	200 (37.7)	<.001	287 (54.2)	97 (18.3)	146 (27.5)	<.001
No	5626 (91.4)	2439 (43.4)	2450 (43.6)	736 (13.1)		4033 (71.7)	830 (14.8)	763 (13.6)	
Positive family support									
Low positive support	2565 (41.4)	591 (23.0)	1285 (50.1)	689 (26.9)	<.001	1463 (57.0)	517 (20.2)	585 (22.8)	<.001
High positive support	3627 (58.6)	1973 (54.4)	1403 (38.7)	250 (7.0)		2885 (79.5)	415 (11.4)	327 (9.0)	
Negative family interactions									
Low negative interactions	3044 (49.2)	1776 (58.4)	1079 (35.5)	188 (6.2)	<.001	2514 (82.6)	303 (10.0)	227 (7.4)	<.001
High negative interactions	3148 (50.8)	788 (25.0)	1609 (51.1)	751 (24.0)		1834 (58.3)	629 (20.0)	685 (21.8)	
Maladaptive SM use									
No SM/less maladaptive use	3220 (51.9)	1558 (48.4)	1304 (40.5)	357 (11.1)	<.001	2479 (77.0)	412 (12.8)	329 (10.2)	<.001
High maladaptive use	2990 (48.1)	1014 (33.9)	1394 (46.6)	582 (19.5)		1883 (63.0)	522 (17.5)	585 (19.6)	
School connectedness									
Low	2632 (42.3)	525 (20.0)	1364 (51.8)	743 (28.2)	<.001	1290 (49.0)	608 (23.1)	734 (27.9)	<.001
High	3594 (57.7)	2054 (57.2)	1342 (37.4)	197 (5.5)		3084 (85.8)	330 (9.2)	180 (5.0)	
Positive school climate									
Low	2898 (49.6)	767 (26.5)	1441 (49.7)	690 (23.8)	<.001	1726 (59.6)	578 (19.9)	594 (20.5)	<.001
High	2945 (50.4)	1592 (54.1)	1133 (38.5)	219 (7.4)		2350 (79.8)	319 (10.8)	276 (9.4)	

Notes: (1) The P-value is from the Rao-Scott Chi-square test, which is a design-adjusted Pearson Chi-square test for survey data. (2) Family support variables (positive and negative support), maladaptive social media use, school connectedness, and positive school climate variables were categorized as: scores<median for low and scores ≥median for high levels (3) SM refers to social media. No SM and less maladaptive SM use were combined in a single category.

© 2025 Bista S et al. *JAMA Network Open*.
