# Supplementary material for: Depression and Anxiety Among Young Gender- and Sexuality-Diverse Adolescents
Source: JAMA Netw Open. 2025 Dec 29;8(12):e2551570. doi: 10.1001/jamanetworkopen.2025.51570 (PMC12750252; doi:10.1001/jamanetworkopen.2025.51570)
Supplement: Supplement 2. — Data Sharing Statement [file jamanetwopen-e2551570-s002.pdf]

Data Sharing Statement

Bista. Depression and Anxiety Among Young Gender- and Sexuality-Diverse Adolescents. *JAMA Netw Open*. Published December 29, 2025. doi:10.1001/jamanetworkopen.2025.51570

Data
Data available: No

Additional Information
Explanation for why data not available: Due to privacy/ethical restrictions, the data are not publicly available but may be available on request to the Future Proofing Study Committee, Black Dog Institute.
